# Attention-based multi-scale features fusion for unobtrusive atrial fibrillation detection using ballistocardiogram signal

**DOI:** 10.1186/s12938-021-00848-w

**Published:** 2021-01-28

**Authors:** Fangfang Jiang, Chuhang Hong, Tianqing Cheng, Haoqian Wang, Bowen Xu, Biyong Zhang

**Affiliations:** 1 College of Medicine and Biological Information Engineering, Northeastern University, Shenyang, China; 2 College of Medicine and Biological Information Engineering, Eindhoven University of technology, Eindhoven, The Netherlands; 3 BOBO Technology, Hangzhou, China

**Keywords:** Ballistocardiogram signal, Phase space reconstruction, Bi-LSTM, CNN, Feature fusion, Attention mechanism

## Abstract

**Background:**

Atrial fibrillation (AF) represents the most common arrhythmia worldwide, related to increased risk of ischemic stroke or systemic embolism. It is critical to screen and diagnose AF for the benefits of better cardiovascular health in lifetime. The ECG-based AF detection, the gold standard in clinical care, has been restricted by the need to attach electrodes on the body surface. Recently, ballistocardiogram (BCG) has been investigated for AF diagnosis, which is an unobstructive and convenient technique to monitor heart activity in daily life. However, here is a lack of high-dimension representation and deep learning analysis of BCG.

**Method:**

Therefore, this paper proposes an attention-based multi-scale features fusion method by using BCG signal. The 1-D morphology feature extracted from Bi-LSTM network and 2-D rhythm feature extracted from reconstructed phase space are integrated by means of CNN network to improve the robustness of AF detection. To the best of our knowledge, this is the first study where the phase space trajectory of BCG is conducted.

**Results:**

2000 segments (AF and NAF) of BCG signals were collected from 59 volunteers suffering from paroxysmal AF in this survey. Compared to the classical time and frequency features and the state-of-the-art energy features with the popular machine learning classifiers, AF detection performance of the proposed method is superior, which has 0.947 accuracy, 0.935 specificity, 0.959 sensitivity, and 0.937 precision, for the same BCG dataset. The experimental results show that combined feature could excavate more potential characteristics, and the attention mechanism could enhance the pertinence for AF recognition.

**Conclusions:**

The proposed method can provide an innovative solution to capture the diverse scale descriptions of BCG and explore ways to involve the deep learning method to accurately screen AF in routine life.

## Background

Recently, the lifetime risks of atrial fibrillation (AF), one kind of arrhythmias, have been estimated in individuals of European ancestry from $\approx$ 1 in 4 increased to $\approx$ 1 in 3 [1, 2]. In a medical insurance database study from the Yunnan Province in China, the estimated lifetime risk of AF at age 55 years was 21.1$\%$ for females and 16.7$\%$ for males [3]. Investigators from the NHLBI-sponsored ARIC study observed that the lifetime risk of AF was 36$\%$ in white males, 30$\%$ in white females, 21$\%$ in African American males, and 22$\%$ in African American females [4]. Overall data show the urgency and necessity of AF monitoring in daily life.

Electrocardiogram (ECG) is recognized as the gold standard for detecting AF in clinical care, with P wave vanished and heartbeat rhythm dysrhythmia. However, ECG diagnosis relies on the electrode adhered to the skin, the costly monitor, and the medical expert guide. Due to the characteristics of AF, such as sudden onset, high recurrence, etc., recent advances in AF diagnosis have created new challenges to the unobstructive measurement and automatic detection during daily life. Ballistocardiogram (BCG) records the weak vibration signal on the surface of the body transmitted by the cardio-dynamic force, which is utilized to estimate the cardiac function. BCG waveform incorporates diverse shape, amplitude, and rhythm, and the irregular BCG may reveal abnormal circulation and cardiac diseases. Its general measuring hardware is multitudinous, for instance, the modified weighing scale, chair, mattress, force platform, even a camera [5]. On account of the superiorities mentioned above, BCG is increasingly applied to automatically monitor heart diseases at home.

### A. Related works

Several recent studies have demonstrated the feasibility of diagnosing AF by using BCG signal. Bruser extracted 17 statistical features of the 30-s BCG segment in time and time-frequency domain, and applied seven popular machine learning (ML) algorithms to separate BCG signals into three classes: sinus rhythm (SR), AF, and artifact [6]. Zink detected the heartbeat cycle length with BCG and examined the correlation of the heartbeat characteristics between BCG and synchronized ECG, which illustrated the feasibility of distinguishing SR and AF [7]. Recently, Yu split the BCG records during sleep into 30-s segments, extracted the stationary wavelet transform features and utilized three popular ML classifiers to automatically detect AF [8]. In Wen’s study, BCG signals were split into 1-min segments and transformed to the energy signals, from which four data sequences representing different characteristics were generated and 16 features were extracted, then five ML algorithms were used for identifying AF and SR [9].

The primary research procedures of the aforementioned methods usually include segmentation, feature extraction, feature selection, and classification. Among them, the feature extraction and selection methods were crucial, including the time feature, frequency feature, and time-frequency feature, which relied on the peaks and the troughs of the BCG waveform. In addition, most previous research applied the ML classifiers, such as the support vector machine (SVM), Naive Bayes (NB), decision tree (DT), bootstrap aggregated decision trees (BAT), random forests (RF) and so on, required to match the corresponding features to obtain the satisfied AF classification accuracy. Therefore, the main challenge of the existing methods is how to extract reliable features from diverse BCG waveforms, which will directly determine the AF classification performance.

To avoid the dependence on the BCG morphology, we attempt to apply deep learning (DL) method to detect AF, which has been successfully occupied to classify the ECG signal. The DL algorithms involve both the feature extraction and the classification in the training process, especially the feature extraction and selection are accomplished adaptively. For this condition, numerous successful cases with respect to the AF detection automatically based on ECG signal by means of deep neural networks have been implemented. For example, Wu proposed a novel approach based on deep belief networks (DBN) for features learning of ECG arrhythmias [10]. He proposed a new method for automatic classification of arrhythmias using the combination of deep residual network (DRN) and bidirectional long short-term memory (Bi-LSTM) network [11]. Fan proposed a multi-scaled deep convolutional neural network (CNN) fusion method to screen out AF recordings from single lead short ECG recordings, which employ the architecture of two-stream convolutional networks with different filter sizes to capture features of different scales [12]. Following the aforementioned successful examples in ECG analysis, we have applied the transfer learning principle to design an effective CNN framework for AF detection by means of BCG signal [13].

### B. Contributions

In this work, we sought to employ the 1-D Bi-LSTM and phase space reconstruction (PSR) algorithm to severally represent the 1-D morphology feature and 2-D rhythms feature of BCG signal. In addition, an integrated framework based on CNN with attention mechanism is proposed to further improve the AF classification performance. In particular, the main contributions of this work are: (1) to the best of our knowledge, this paper is the first to apply the Bi-LSTM model to extract the features from one single BCG heartbeat. We designed the structure of the network and adjusted the parameters. (2) This paper for the first time utilizes the PSR theory to extract the rhythms feature of BCG signal. We draw the phase space trajectory and verified the susceptibility to the disordered rhythm. (3) The attention mechanism is involved to assist the integration of the two features with different dimensions. To obtain the eminent classification accuracy and pervasiveness, we attempt two ways of attention mechanism modules and optimize the final solution. In order to evaluate the performance of proposed method, we implemented the classical features in [6] and the up-to-date energy features in [9] with five popular ML classifiers to the same BCG dataset as a comparison.

The results and detailed descriptions will be organized as follows: first, describe the experimental procedure and introduce the data distribution; second, introduce and illustrate the performance of the proposed method; third, re-implement the previous methods with the same BCG dataset; fourth, discuss and conclude the results, and finally, provide the details of the multi-scale features extraction method and the integrated framework with attention mechanism.

## Results

### A. Experimental procedure

**Fig. 1:**
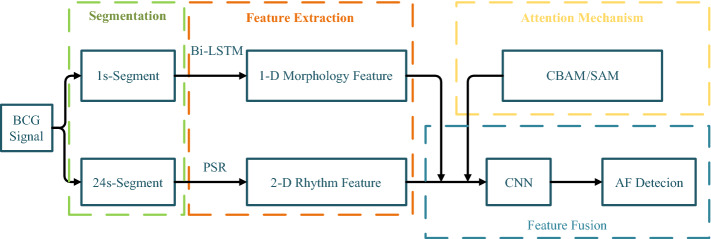
Modular framework of the proposed method for AF detection by means of BCG signal. First, the AF and NAF signals were split into the 1-s-segment dataset and the 24-s-segment dataset, respectively. Second, the 1-D morphology feature extracted from Bi-LSTM and the 2-D rhythms feature extracted from PSR were integrated by CNN. Ultimately, the attention mechanism was involved to improve the performance of the AF classification

For the purpose of screening AF from BCG signal in the routine life, we added Bi-LSTM network, which is successfully applied to AF detection from ECG signal, to the CNN network we designed and verified in our previous work [13]. Figure 1 illustrates a block diagram of the proposed method, which consists of multi-dimension features extraction and integrated framework based on attention mechanism.

In order to validate the performance of proposed method, we designed the experimental procedure based on the methodological steps. First, the experimental data collection, segmentation and distribution were introduced. Second, the AF classification performance of the proposed method was demonstrated. In this section, the effects of the single features, the integrated feature and the attention mechanism were presented, respectively. Third, two previous methods were implemented by means of the same BCG dataset in this survey as a comparison. The 17 classical time and time-frequency features were extracted and classified by 5 popular ML algorithms based on [6]. And the BCG energy signal and the 16 statistic features were calculated and fed into the 5 popular ML classifiers based on [9]. This step is aimed to verify the superiority of the proposed method.

In the comparison process, the performance parameters including accuracy(Acc), sensitivity(Sen), precision(Pre), and specificity(Spe) were interpreted and calculated as Formula (1)–(4) in accordance to the confusion matrix:

1 $$\begin{aligned} \text {Acc}=(\text {TP}+\text {TN})/(\text {TP}+\text {FP}+\text {TN}+\text {FN),} \end{aligned}$$

2 $$\begin{aligned} \text {Sen} ={\rm TP}/({\rm TP}+{\rm FN}), \end{aligned}$$

3 $$\begin{aligned} \text { Pre} ={\rm TP}/({\rm TP}+{\rm FP}),\end{aligned}$$

4 $$\begin{aligned} \text {Spe} = {\rm TN}/({\rm TN}+{\rm FP}),\end{aligned}$$

where TP is the number of true positives, TN is the number of true negatives, FP is the number of false positives, and FN is the number of false negatives.

### B. Data collection and distribution

Fifty nine volunteers suffering from paroxysmal AF (34 males and 25 females), ranging in age from 27 to 93 years, participated in this study. For each subject, the synchronous BCG and ECG data were recorded for 8 h, in the lying position from 0 a.m. to 8 a.m. In this process, the acquisition instruments and the signal preprocessing method will be detailed in the “Method” section. After obtaining the pure BCG and ECG signals, the AF period and non-atrial fibrillation (NAF) period of BCG signal were labeled manually by medical experts according to the synchronous ECG signal as a reference.

**Fig. 2:**
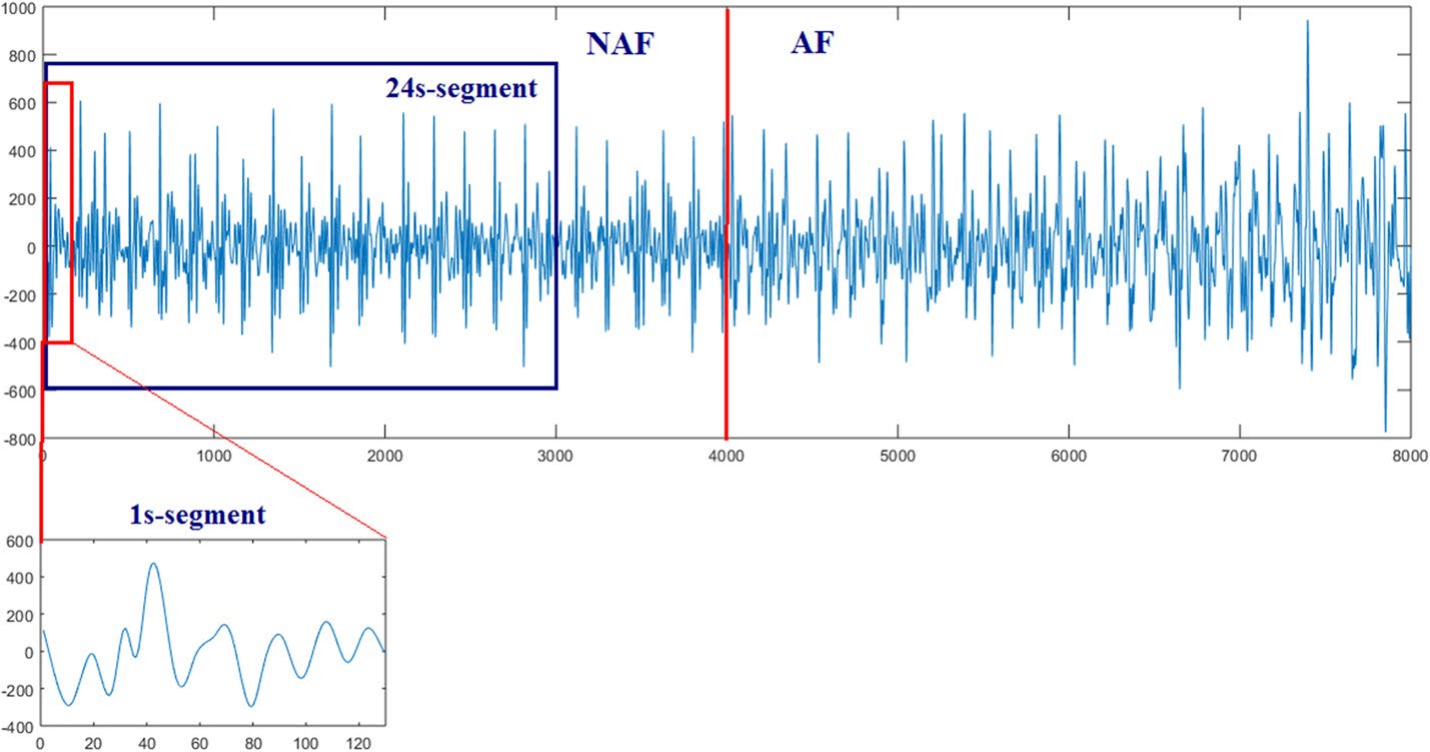
AF and NAF BCG signal: 1-s-segment and 24-s-segment are illustrated. The left part in this waveform is labeled as AF period, the right part is labeled as NAF period, and all periods were split manually by medical experts in accordance to the synchronous ECG signal as a reference. In Fig. 2, the 24-s-segment is divided from the original BCG signal, and the 1-s-segment is extracted from the 24-s-segment by means of taking 0.5 s before and 0.5 s after the occurrence of the J-peak in BCG waveform

In order to extract the multi-scale features in the next step, the 1-s-segment and 24-s-segment were selected, respectively, on the basis of previous literatures [12–16]. Firstly, the entire AF and NAF periods were segmented as 24-s-segment without overlap. And then 1-s-segment was extracted from the corresponding 24-s-segment to uniform the amount of datasets. In each 24-s-segment, take 0.5 s before and 0.5 s after the occurrence of the J-peak in BCG as 1-s-segment. The processing of data segmentation is illustrated in Fig. 2.

From the above, a total of 2000 BCG segments (1000 labeled AF and 1000 labeled NAF) were obtained in both 1-s-segment dataset and 24-s-segment dataset. Among them, 80$\%$ of the original dataset is applied to train the network, which is divided into training dataset and validation dataset with a ratio of 4:1, and the remaining 20$\%$ of the original dataset is recognized as independent testing dataset. In order to ensure the fairness of the experiments, all segments were collected from 59 subjects as evenly as possible, and the training dataset and the testing dataset derived from different subjects to avoid overfitting.

**Table 1:** Confusion matrix of the proposed method

Method	Classify results	True condition	
		AF	SR
Bi-LSTM	AF	752	115
	SR	248	885
DBN	AF	710	266
	SR	290	734
PSR-CNN	AF	935	102
	SR	65	898
FF-CNN	AF	953	94
	SR	47	906
FF-CNN-SAM	AF	949	89
	SR	51	911
*FF-CNN-CBAM*	AF	*959*	*65*
	SR	*41*	*935*

### C. Results of the proposed method

In this section, we compared the AF classification performance of the single features by means of the Bi-LSTM, DBN and CNN, respectively. And then the effects of the integrated feature and the attention mechanism were presented, respectively. In this process, the tenfold cross-validation was utilized to evaluate the performance of the classification algorithms, which could avoid inaccurate assessments in extreme situations [17]. The confusion matrix is represented in Table 1, and the performance parameters are shown in Table 2.

The results of the 1-D morphology feature extracted from the Bi-LSTM are shown in row 1 of Tables 1 and 2, denoted as Bi-LSTM. In the previous work, we have successfully detected AF from BCG signal by means of CNN [13]. In this work, we employed Bi-LSTM to extract the 1-D morphology feature from 1-s-segment BCG dataset. Bidirectional LSTM (Bi-LSTM) is an improved version of LSTM, which is availably applied to detect AF from ECG signal [11]. And the details of the algorithm are introduced in the "Method" section B.

As a comparison, the results of the similar 1-D neural network DBN is shown in row 2 of Tables 1 and 2, denoted as DBN. DBN has been successfully applied to detect AF from ECG signal [10]. In this survey, we employed DBN to extract the 1-D morphology feature from 1-s-segment BCG dataset. This step is aimed to select the appropriate 1-D neural network to classify the 1-s-segment BCG dataset.

The results of the 2-D rhythms feature extracted from PSR and CNN are shown in row 3 of Tables 1 and 2, denoted as PSR-CNN. The 2-D rhythms feature, which were extracted by the phase space trajectory of 24-s-segment BCG dataset, was fed into the designed CNN framework [13]. PSR is a mathematical method used to analyze complex systems, which maps 1-D time series to the high-dimensional space via a constructor [18]. And the details of the algorithm are introduced in the "Method" section C.

On the basis of the single features given above, the results of the feature fusion are shown in row 4 of Tables 1 and 2, denoted as FF-CNN. The 1-D morphology feature extracted from Bi-LSTM and the 2-D rhythm feature extracted from PSR and CNN achieved the superior performance. Therefore, we attempted to integrate the features to further improve the AF detection performance. And the integrated framework based on CNN and Bi-LSTM is elaborated in the "Method" section D.

In addition, we also explored to, respectively, involve the attention mechanism layer SAM and CBAM to the integrated framework to improve the accuracy. The results are shown in row 5 and row 6 of Tables 1 and 2, denoted as FF-CNN-SAM and FF-CNN-CBAM. And the details of the two ways of attention mechanisms are described in the "Method" section E. This step is aimed to determine whether the addition of the attention mechanism improve the accuracy and to select the appropriate attention mechanism module for the integrated framework.

### D. Results of the previous methods

**Table 2:** Classification performance of the proposed method

Method	ACC	SPE	SEN	PRE
Bi-LSTM	0.819	0.885	0.752	0.867
DBN	0.722	0.734	0.710	0.728
PSR-CNN	0.917	0.898	0.935	0.902
FF-CNN	0.929	0.906	0.953	0.910
FF-CNN-SAM	0.930	0.911	0.949	0.914
*FF-CNN-CBAM*	*0.947*	*0.935*	*0.959*	*0.937*

As a comparison, the traditional AF detection method from BCG signal was implemented in accordance with the previous study on [6]. 17 classical time and time-frequency features (6 time domain and 11 time-frequency domain) were extracted from the same BCG dataset in this survey. And then, 5 popular ML models were utilized to classify AF and NAF, including SVM, NB, BAT, RF, and DT. The selection and implementation of the ML algorithms were based on [9, 19, 20]. The confusion matrix is represented in Table 3, and the performance parameters are shown in Table 4.

**Table 3:** Confusion matrix of the previous method [6]

Method	Classify results	True condition	
		AF	SR
SVM	AF	645	278
	SR	355	722
NB	AF	454	141
	SR	546	859
BAT	AF	780	214
	SR	220	786
*RF*	AF	*858*	*221*
	SR	*142*	*779*
DT	AF	746	231
	SR	254	769

**Table 4:** Classification performance of the previous method [6]

Method	ACC	SPE	SEN	PRE
SVM	0.684	0.722	0.645	0.699
NB	0.657	0.859	0.454	0.763
BAT	0.783	0.786	0.780	0.785
*RF*	*0.819*	*0.779*	*0.858*	*0.795*
DT	0.758	0.769	0.746	0.764

In addition, the up-to-date AF detection method by means of BCG signal was implemented based on [9]. In [9], BCG signals were transformed into BCG energy signals and 4 new data sequences representing different characteristics of the BCG energy signals were generated. The mean value, variance, skewness, and kurtosis of the 4 data sequences were calculated and 16 features were extracted for each segment. And 5 popular ML algorithms were used for classification. In this work, we achieved BCG energy signals and data sequences, extracted 16 features, and applied 5 ML classifiers to diagnose AF. The confusion matrix is represented in Table 5, and the performance parameters are shown in Table 6.

**Table 5:** Confusion matrix of the previous method [9]

Method	Classify results	True condition	
		AF	SR
SVM	AF	796	70
	SR	204	930
NB	AF	749	125
	SR	251	875
BAT	AF	845	93
	SR	155	907
*RF*	AF	*846*	*32*
	SR	*154*	*968*
DT	AF	879	100
	SR	121	900

**Table 6:** Classification performance of the previous method [9]

Method	ACC	SPE	SEN	PRE
SVM	0.863	0.930	0.796	0.919
NB	0.812	0.875	0.749	0.857
BAT	0.876	0.907	0.845	0.901
*RF*	*0.907*	*0.968*	*0.846*	*0.964*
DT	0.889	0.900	0.879	0.898

**Fig. 3:**
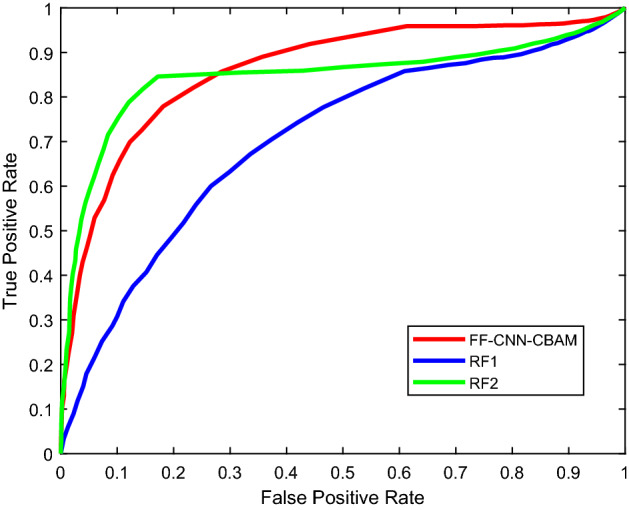
The ROC curves of the proposed method and the superior ML models in previous method [6] and [9]. The red curve represents the classification performance of the proposed method, the blue curve represents the classification performance of the previous method [6] with the superior RF classifier, and the green curve represents the classification performance of the previous method [9] with the superior RF classifier

For evaluating the proposed algorithm quantificationally, the ROC curves of the proposed method and the superior ML models in previous method [6] and [9] are illustrated in Fig. 3. And the area under curve (AUC) of the three curves were calculated, which is 0.733, 0.667, 0.686, respectively.

For evaluating the computational cost of the proposed method, we compared the computational times of the proposed method and the superior ML models in previous method [6] and [9]. We executed the proposed AF classification algorithm in Python without GPU on a computer with Intel (R) Pentium (R) Gold G5500 CPU at 16 GHz, and 128 GB of memory. The operating system was Windows 10 professional 64 bit. We implemented the two previous methods [6] and [9] using MATLAB 2019a. For the proposed method, the computational time needed to train the integrated framework was about 3 h and 47 min, and the mean time to classify the test dataset was 0.78 s. For the previous method [6], the mean time to extract features was 1.24 s, and the mean time to classify the test dataset with RF was 0.63 s. For the previous method [9], the mean time to extract features was 2.09 s, and the mean time to classify the test dataset with RF was 0.75 s.

## Discussion

The previous work on the AF detection based on BCG signal mostly extracted the diverse features of the BCG waveforms, and then classified the features as AF or NAF by means of the popular ML methods [6, 9]. However, the feature extraction absolutely depended on the BCG morphology, which directly determined the classification performance of the ML. Therefore, we attempted to classify the BCG signal with DL method, which implemented the feature extraction and classification synchronously based on supervised autonomous learning. The entire segment was fed into the DL network to achieve superior results. Based on the experimental results, we obtained the following conclusions.

### A. Proposed method analysis

In order to select the appropriate DL network, we compared the single DL networks Bi-LSTM, DBN, and CNN, which have been successfully used to detect AF from ECG signal in [10–12]. Furthermore, we employed CNN to classify AF from BCG signal in [13]. Therefore, the classification performance of these DL networks is compared in Tables 1 and 2. For the 1-s-segment BCG, the Bi-LSTM network is superior to the classical DBN network. This may be because that the Bi-LSTM structure learns the bidirectional dependencies between time steps, which is suitable to analyze 1D time series. Additionally, Bi-LSTM network identifies the subtle distinction between AF and NAF waveform, similar to the P wave disappearance in ECG waveform of AF. Thereby, the BI-LSTM is selected to extract the 1-D morphology feature of BCG segment. For the 24-s-segment BCG, CNN achieved the optimal performance compared with the other DL networks. It is not only related to the longer segment, but also concerned with the input of CNN. In this manuscript, the 1-D BCG segment is reconstructed in 3-D phase space and projected on the 2-D plane, which is proposed as the 2-D rhythm feature. The PSR represents the high-dimension features of BCG, and the CNN is adept in image recognition. Therefore, the combination of PSR and CNN obtained the best classification performance.

For the purpose of performance optimization, we combined the two superior networks and involved the attention mechanism. From Tables 1 and 2, the performance of the integrated framework, which was designed to combine the Bi-LSTM and CNN, is superior to the single PSR-CNN. Additionally, the attention mechanism could facilitate the training process to concentrate on the labels, and improve the performance of ECG classification [21]. Thereby, two attention mechanism modules were added to the integrated framework. By comparing the FF-CNN, FF-CNN-SAM, and FF-CNN-CBAM, the attention mechanism is validated to be conducive to improve classification performance, especially the CBAM module. And the integrated framework with CBAM achieves the optimal performance, with the accuracy, specificity, sensitivity, and precision of 0.947, 0.935, 0.959, 0.937, respectively. Thus, the fusion method and the attention mechanism improve the classification efficiency and robustness.

### B. Previous methods comparison

As a comparison, we extracted the classical time and time-frequency features in [6], and the up-to-date energy features in [9] from the same BCG dataset in this survey. And 5 popular ML classifiers were implemented.

Based on [6], 17 classical time and time-frequency features were extracted, and the classification performance from 5 popular ML classifiers is shown in Tables 3 and 4. The RF classifier performs better than the other four ML classifiers by using the same BCG dataset in this survey, with the accuracy, specificity, sensitivity, and precision of 0.819, 0.779, 0.858, 0.795, respectively. This conclusion is in keeping with [6], but the absolute accuracy value of the optimal RF classifier is lower than [6]. This is related to the diverse waveforms and different SNR of original BCG signals recorded from different equipments. In addition, the amount of the BCG dataset in this survey (2000 segments) was balanced and larger than [6], which occupied 856 segments and unbalanced. The increased data amount and balanced data distribution are orientated to the routine AF screening. In addition, compared with Tables 1 and 2, the classification performance of DL networks was generally superior to the classical time and time-frequency features with ML methods. It is proven that the supervised training is more appropriate to classify the BCG segments compared with the absolute characteristic values, which merely relied on the BCG waveform.

Based on [9], the BCG segment was transformed into energy signal, four new data sequences were generated, and 16 features were extracted for each segment. The classification performance from 5 popular ML classifiers is shown in Tables 5 and 6. For the data volume, [9] and this manuscript utilized the similar BCG datasets. 37 subjects were split into 2915 segments in [9], and 59 subjects were split into 2000 segments in this survey. The former applied more segments, and the latter occupied more subjects. Both of these two BCG datasets were balanced to avoid bias toward the category with more data. For the classification performance, the optimal classifier of [9] implemented by the same BCG dataset in this survey is also RF, with the accuracy, specificity, sensitivity, and precision of 0.907, 0.968, 0.846, 0.964, respectively. It follows that the AF diagnosing performance of the method [9] is superior to the method [6], and the proposed method achieved the optimal classification performance for the same BCG dataset in this survey. This may be because the improved features in [9] promote the AF identification than classical time and time-frequency features in [6]. That means the quality of the feature extraction determines the classification performance. Therefore, this manuscript applied the DL algorithm to extract features and classify AF segments in a supervised manner automatically, which achieved the optimal accuracy. It follows that the DL networks are more applicable to analyze the diverse BCG waveforms from different subjects, such as AF screening.

In terms of the ROC curve, the AUC of the proposed method is larger than the previous methods [6] and [9]. It means that the authenticity of the proposed method is optimum. In terms of the computational time, the training duration of the proposed DL method is longer than the previous ML methods obviously. Nevertheless, the testing duration of the proposed method is approximate to the previous methods, which range from 0.6 s to 0.8 s and satisfy the demand for AF detection in daily life.

### C. Future work

The purpose of this work is to apply the DL networks, which were successfully utilized to detect AF from ECG signal, to identify the AF segment from the BCG signal for the routine screening. By comparison with the same BCG dataset, the DL method proposed in this survey is superior to the traditional ML algorithms. Therefore, we will explore and compare the other deep neuronal networks to classify BCG segments, especially the networks occupied in the ECG signal processing. Hopefully, the comparison results can provide references for the DL network selection in AF detection with BCG signal.

Moreover, we will increase the subjects and the data volume in future work to improve the universality of the algorithm. In addition, we will collect the BCG signals with different postures, for example, the standing and the sitting postures. And the different sensors will be also applied to acquire the raw BCG signal. It is well known that the waveforms of the BCG signals from different instruments are various. Thereby, the DL algorithms will be more effective than the absolute characteristic parameters with ML for the AF detection from different subjects and different instruments.

## Conclusion

This paper demonstrated the great potential for the phase space reconstruction of BCG signal. The phase space trajectory expands 1-D time sequence to 3-D chaotic system, and 2-D tangent plane is trained and tested with CNN, which performs grid-like topology features with less computation. Note that, the optimal model parameters *m* and $\tau$ were firstly discussed and utilized in BCG signal. In addition, the single cycle BCG is fed into Bi-LSTM, due to the inherent instinct for time sequence classification. The assistance of attention mechanism avoids the information redundancy as well as improving the accuracy and robustness. The traditional time and frequency features with ML algorithms and the up-to-date AF classification method were compared with the proposed method with DL. The proposed method obtained the optimal performance for the same BCG dataset in this survey, which proves the superiority of DL algorithm in AF detection by means of BCG signal. In future work, we will implement the proposed method to the different BCG signals, which will be collected from different postures, diverse acquisition equipments and various subjects, to validate the versatility and practicability in AF routine screening.

## Methods

### A. Signal acquisition and preprocessing

BCG is a non-intrusive measurement of the vibration of the body in response to the heartbeat and arterial aortic blood circulation, which is homologous with ECG signal. In this study, a BCG system consisting of the piezoelectric film sensor made of polyvinylidene fluoride (PVDF), set under the bed mattress, was developed to acquire BCG signal with the sampling rate 125 Hz [13]. During the recording process, the raw BCG signal was amplified, filtered by a Butterworth bandpass filter (0.7–10 Hz) to remove the respiratory components, and digitized using a set of signal acquisition hardware circuits with 12-bit resolution. Simultaneously, ECG signal was acquired by the CT-08S dynamic ECG recorder with a sampling rate 200 Hz. In order to address the different sampling rates with BCG signal, ECG signal was downsampled to 125 Hz based on the synchronous timestamp.

To achieve the pure BCG signal, the periods of “out of bed” and motion artifacts were eliminated based on the excessively large or small amplitude and the variations of the BCG signal’s envelope. After that, each pure BCG signal was normalized with its maximum and minimum value. This operation is aimed to reduce the impact of different amplitude levels, which is possibly derived from different age, height, weight, sleep postures, and so on. Because the method proposed is intended to screen AF in routine life, the influence of individual factors was minimized to improve the generality of the algorithm. Ultimately, the remaining periods of BCG signal were labeled as AF and NAF periods, which were further split as 1-s and 24-s segments, respectively.

### B. 1-D morphology feature based on Bi-LSTM

Long short-term memory (LSTM) network was firstly introduced in 1997 by Hochreiter to address the exploding and vanishing gradient problems [22]. LSTM performs well in dealing with tasks involving sequence classification, e.g., speech recognition. Bidirectional LSTM (Bi-LSTM) is an improved version of LSTM. Given a time series as input, the network is able to capture the features from each time step in both forward and backward directions. The output features can be fed into other networks as indicators for classification or forecasting. In the field of biomedical engineering, LSTM has been proven to be effective in bio-signals processing: EEG classification [23], and ECG classification [24].

**Fig. 4:**
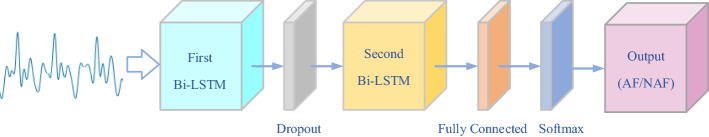
Structure of the Bi-LSTM network, which is applied to extract 1-D morphology feature. Both Bi-LSTM layers comprised 50 hidden units, to match the 1-s-segment input. The first Bi-LSTM layer reads the data fed from the input layer and outputs a complete sequence at each time step. The second one is configured to only output at the last time step of the sequence, which can be treated as a feature vector for classification. An additional dropout layer with a probability of 0.5 is inserted between the layers to avoid overfitting, ensuring the trained model’s ability of generalization. The output feature vector from the second Bi-LSTM layer is fed into a fully connected layer, which maps the features from 50 dimensions to 2 dimensions. A Softmax function is then employed to the 2-D vector and the followed classification layer gives an AF or NAF result. The initial learning rate is set as 0.0005 and the network training takes 30 epochs

According to the homology between ECG and BCG, we firstly attempt to detect AF from BCG signal with Bi-LSTM. The network is composed of two Bi-LSTM layers, one dropout layer, one fully connected layer, and one Softmax layer. The network structure is illustrated in Fig. 4, and the network parameters are listed in Table 7.

**Table 7:** Network parameters of the Bi-LSTM

Layer	Size	Description
Input	125	Input signal length
Bi-LSTM	50	First Bi-LSTM layer
Dropout	50	Random dropout to avoid overfitting
Bi-LSTM	50	Second Bi-LSTM layer
FC	2	Map the features
Softmax	2	Softmax function
Output	2	Output the classification results

The network is trained by 1-s-segment BCG dataset, and the output of the second Bi-LSTM layer is denoted as 1-D morphology feature, which contains abundant temporal information [25].

### C. 2-D rhythm feature based on phase space reconstruction

Phase space reconstruction (PSR) is a mathematical method used to analyze complex systems, which maps 1-D sampled data to high-dimensional space via a constructor [21]. Recently, PSR has been successfully involved to extract key features from the blood pressure and ECG signal [26–28], due to its visualization and quantification of changes in particular features of the interval lengths. Considering the same rhythm between ECG and BCG, this paper generates a reconstructed attractor in 3-D phase space and projects the attractor onto a plane from 24-s-segment BCG data to achieve the 2-D rhythm feature.

Our method consists of three steps:

1. Determine the embedding dimension *m* and the time delay parameter $\tau$.
2. Reconstruct an attractor using delay coordinates.
3. Remove the baseline variation and obtain the 2-D rhythm feature.

In this procedure, it is vital to choose the appropriate parameters *m* and $\tau$.

With regard to the embedding dimension *m*, various methods have been proposed to determine the minimum dimension during the reconstruction of the phase space of a dynamical system, including a singular value analysis, the false nearest neighbors, Cao’s method, or empirically [29–31]. In this manuscript, we utilize the false nearest neighbors method to determine the optimal embedding dimension *m*, which is regarded as the most popular method. Its theory is to find a negligible number of false neighbors while the dimension is added from *m* to *m*+1, during the neighbors are checked with increasing embedding dimensions. We calculated the Euclidean distance between neighbors and confirmed the optimal embedding dimension *m* is 3 for 24-s-segment BCG signal.

With regard to the time delay parameter $\tau$, if $\tau$ is too small, the difference between the variables will be subtle; if $\tau$ is too large, there will be little correlation between each of the variables. Usually, the first minimum of the average mutual information function, the first zero crossing of the autocorrelation function, or the empirical method is occupied to determine $\tau$ in a middle range. In this manuscript, the autocorrelation function method is applied to select the appropriate $\tau$ [32]. After calculating, find the first zero crossing point of the autocorrelation function and confirm the optimal time delay parameter $\tau$ is 40 ms for 24-s-segment BCG signal.

After choosing *m* = 3, and $\tau$ = 40ms, an attractor is reconstructed in the 3-D phase space by means of Takens delay coordinates [18]. Suppose the 24-s-segment BCG signal is *x*(*t*), then the two new variables *y* and *z* are defined as Formula (5) and (6):

5 $$\begin{aligned} y (t )\,=\,& {} x (t -\tau ), \end{aligned}$$

6 $$\begin{aligned} z (t )= x (t -2\tau ). \end{aligned}$$

Then the vector (*x*(*t*), *y*(*t*), *z*(*t*)) can plot the 3-D trajectory in the reconstructed phase space for the variable *t* in 24-s-segment. To avoid the change of the baseline for each segment, projecting the 3-D attractor onto a plane is usually applied to eliminate the effect of a constant vertical translation [32]. The three new variables *u*, *v*, *w* are defined as Formula (7)–(9):

7 $$\begin{aligned} u= & {} \frac{1}{\sqrt{3}}(x +y +z ), \end{aligned}$$

8 $$\begin{aligned} v= & {} \frac{1}{\sqrt{6}}(x +y -2z ), \end{aligned}$$

9 $$\begin{aligned} w= & {} \frac{1}{\sqrt{2}}(x -y ). \end{aligned}$$

**Fig. 5:**
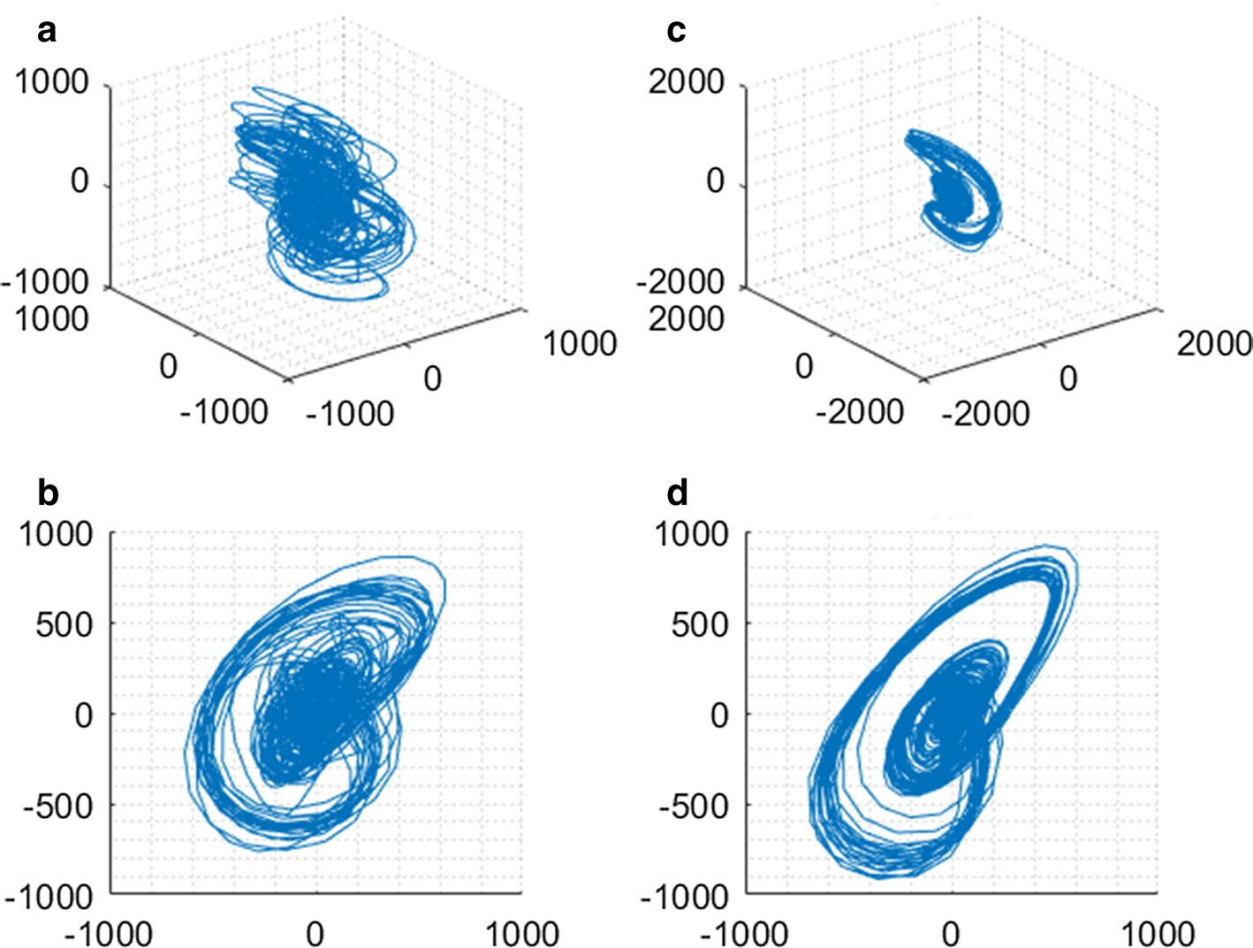
Phase space reconstruction of AF and NAF. **a**, **c** are the 3-D trajectory of AF and NAF, respectively; **b** and **d** are the 2-D projecting plant of AF and NAF, respectively. The 2-D projection is denoted as the 2-D rhythm feature, which represents the different variation of the intervals between AF and NAF. And the overall 2-D projection plot will be used as the input of the deep neural network

This transformation can coordinate the reconstructed phase space in the direction of the vector (1, 1, 1), and keep the shape of the trajectory consistent. Each point in the trajectory describes a potential state of the dynamic system, therefore, the 2-D projection is denoted as the 2-D rhythm feature. Figure 5 illustrates the 3-D trajectory in reconstructed phase space and the 2-D projection for one NAF and one AF segment.

As shown in Fig. 5, the 2-D projection presents more obvious rhythmic variation than the 3-D phase space trajectory. In addition, the 2-D projection of AF appears more chaotic components than NAF, which is difficult to quantify the subtle distinction. Therefore, the entire plot of the 2-D projection is regarded as the 2-D rhythm feature based on BCG signal for AF detection.

### D. Integrated framework

For combining the diverse dimensional features, an integrated framework based on convolutional neural network (CNN) is designed, which imitates the visual perception mechanism of the organism. CNN is a feed-forward neural network with deep structure and convolution computation. The sharing of convolution kernel parameters in the hidden layer and the sparsity of interlayer connections provide the grid-like topology features with less computation. Recently, CNN has been successfully employed to image recognition and medical image classification, which enable data-driven learning, high representation and hierarchical image features. Therefore, in this work, extracting the features of phase space trajectory and integrating the diverse scale features are accomplished on the CNN framework.

The designed CNN mainly consists of eight convolution layers, four pooling layers, four dropout layers, one flatten layer, one full connection layer, and outputs the result of dichotomy. Among them, the convolution layer extracts and analyzes the high-dimensional features deeply. The pooling layer effectively reduces the feature matrix and parameters to retain the information and avoid overfitting. Dropout layer increases the anti-overfitting ability of CNN. The flatten layer compresses the feature and outputs as 1-D feature vector. The network parameters are listed in Table 8. And we have demonstrated the effectiveness of the proposed CNN structure in [13], which showed superior performance for AF diagnosing via BCG signal.

**Table 8:** CNN parameters of the convolution layers and the pooling layers

Layer number	Number of convolution kernels	Kernel size
1	32	$9 \times 1$
2	32	$9 \times 1$
3	3	$3 \times 1$
4	64	$9 \times 1$
5	64	$9 \times 1$
6	3	$3 \times 1$
7	128	$9 \times 1$
8	128	$9 \times 1$
9	3	$3 \times 1$
10	256	$9 \times 1$
11	256	$9 \times 1$
12	3	$3 \times 1$

**Fig. 6:**
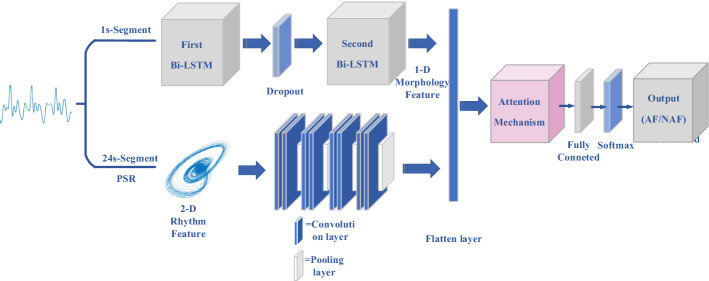
Integrated framework based on CNN and Bi-LSTM. The 1-D morphology feature extracted from Bi-LSTM network by means of 1-s-segment BCG and the 2-D rhythm feature extracted from PSR by means of 24-s-segment BCG are combined by using the CNN framework. In addition, the attention mechanism will be involved to improve the AF classification performance

In addition, on the basis of the CNN structure we designed, 1-D morphology feature extracted from Bi-LSTM network is combined to realize multi-scale features fusion. The integrated framework is illustrated in Fig. 6.

In order to integrate the diverse dimension features, splice the outputs of the flatten layers of Bi-LSTM and CNN, and the full connection layer and outputs of dichotomy keep consistent. Additionally, the attention mechanism layer is added between the Flatten layer and the full connection layer to enhance the fusion performance.

### E. Attention mechanism

Attention modeling is usually employed to improve the performance on the main task in neural networks [33]. In addition, it also helps to solve the performance degradation caused by the increase of input length, as well as the computational inefficiency caused by the sequential processing of input [34]. In this manuscript, the classical self-attention mechanism (SAM) method and the convolutional block attention module (CBAM) method were, respectively, introduced as a comparison.

#### (1) Self-attention mechanism (SAM)

**Fig. 7:**
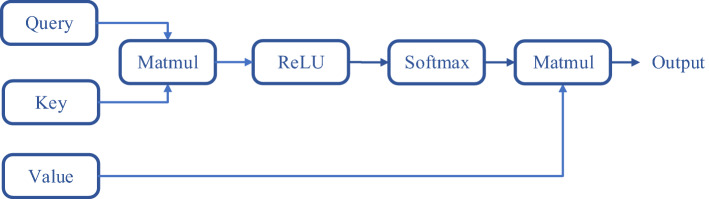
Structure of the SAM layer. The MATMUL function is calculating the matrix multiplication, the ReLU function is used as the activation function and the Softmax function is utilized for training and normalization. The details of the calculation steps are listed in Formula (10)–(15)

Kuvaev proposed an attention model for ECG classification based on a residual attention network consisting of multiple ResNet blocks and slightly modified attention modules [21]. On this basis, we add the SAM layer into the integrated framework in allusion to diagnose AF. The input of this layer consists of the query, value, and key. The output is the weighted sum of the output of the attention-weighted matrix, which is determined by queries and keys. The structure of the SAM layer is illustrated in Fig. 7.

Suppose the length of input vector **X** is L, and take T as the step size to divide **X** into multiple vectors ${\mathbf{X }}_U^t$ (${\text {T}}=\sqrt{\text {L}}$, round up to an integer). By linear projection mapping of the feature vector ${\mathbf{X }}_U^t$, query matrix $\mathbf{Q }'$, key matrix ${\mathbf{K }}'$ and value matrix $\mathbf{V }'$ are obtained based on Formula (10)–(12):

10 $$\begin{aligned} \mathbf{Q }'= \text {ReLU}({\mathbf{X }}_U ^t \mathbf{W }_Q ^t ), \end{aligned}$$

11 $$\begin{aligned} \mathbf{K }'= \text {ReLU}(\mathbf{X }_U ^t \mathbf{W }_K ^t ),\end{aligned}$$

12 $$\begin{aligned} \mathbf{V} '= \text {ReLU}(\mathbf{X }_U ^t \mathbf{W }_V ^t ),\end{aligned}$$

where ${\mathbf{W }}_U^t$, ${\mathbf{W }}_K^t$, ${\mathbf{W }}_V^t$ are linear mappings weights obtained through training, and the ReLU function is used as the activation function to achieve the input query, key and value. After that, the nonlinear representation and Softmax function are utilized for training and normalization. Finally, the attention weight matrix $\mathbf{S }_u$ and the output of SAM layer $\mathbf{A }_u$ are calculated as Formula (13)–(15):

13 $$\begin{aligned} \mathbf{S }_u ^t= {\text {Softmax}}\left(\frac{\mathbf{Q }'{\mathbf{K }}'{\text {T}}}{\sqrt{\text {d}}}\right), \end{aligned}$$

14 $$\begin{aligned} \mathbf{S }_u= \sum _{t =1}^{X/Y}\mathbf{S }_u ^t , \end{aligned}$$

15 $$\begin{aligned} \mathbf{A }_u= \mathbf{S }_u \mathbf{V }', \end{aligned}$$

where dot-product attention and the scaling factor $\sqrt{\text {d}}$ are involved to reduce the influence of gradient. Dot-product attention is faster and space-efficient in practice, due to the highly optimized matrix multiplication code. And the parameter d is set to a constant 100 empirically [35].

#### (2) Convolutional block attention module (CBAM)

Woo proposed a new lightweight attention module CBAM, which could be added to any location of neural network [36]. Similar to the SAM layer, the CBAM layer is added between the flatten layer and the full connection layer in this manuscript. In addition, the CBAM incorporates two sub-modules in sequence: the channel attention module and the spatial attention module.

**Fig. 8:**
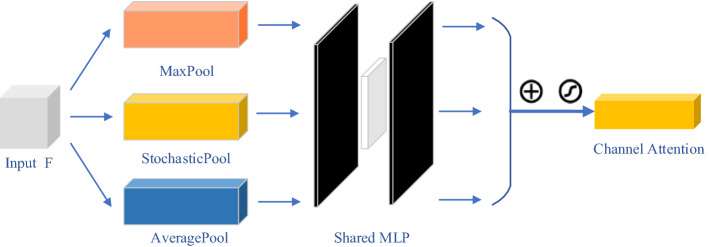
Structure of the channel attention module. The stochastic pool function is supplemented to the general average pool and maximum pool functions together, which effectively improves the presentation capability of the network. And the multilayer perceptron (MLP) is utilized to train the network

*Channel attention module:* the structure of the channel attention module is illustrated in Fig. 8.

The inter-channel relationships of the features are utilized to generate the channel attention, which is focused on “what” makes sense for the given input. Additionally, the stochastic pool function is supplemented to the general average pool and maximum pool functions together, which improve the presentation capability of the network effectively. Suppose the input of the channel attention module is the vector **F**, and the overall procedure can be calculated as Formula (16)–(17).

16 $$\begin{aligned} \text {M}_c ({\mathbf{F }}) &=  \sigma (\text {MLP}(\text {AvgPool}({\mathbf{F }}))+\text {MLP}(\text {MaxPool}({\mathbf{F }}))+\text {MLP}(\text {StoPool}({\mathbf{F }})))\nonumber \\ &= \sigma ({\mathbf{W }}_\text {1}({\mathbf{W }}_\text {0}({\mathbf{F }}_{avg} ^C ))+\mathbf{W }_\text {1}(\mathbf{W }_\text{0 }{}(\mathbf{F }_{max} ^C ))+\mathbf{W }_\text {1}(\mathbf{W }_\text {0}(\mathbf{F }_{sto} ^C ))), \end{aligned}$$

17 $$\begin{aligned} \mathbf{F }'= \text {M}{} c (\mathbf{F })\otimes \mathbf{F ,} \end{aligned}$$

where $\mathbf{W }_0$ and $\mathbf{W }_1$ are the weights of the Multilayer Perceptron (MLP), $\otimes$ is elementwise product, and $\sigma$ is the sigmoid function. $\mathbf{F }_\text {avg}^C$ is the output of the average pooling. $\mathbf{F }_\text {max}^C$ is the output of the max pooling. $\mathbf{F }_\text {sto}^C$ is the output of the stochastic pooling.

**Fig. 9:**
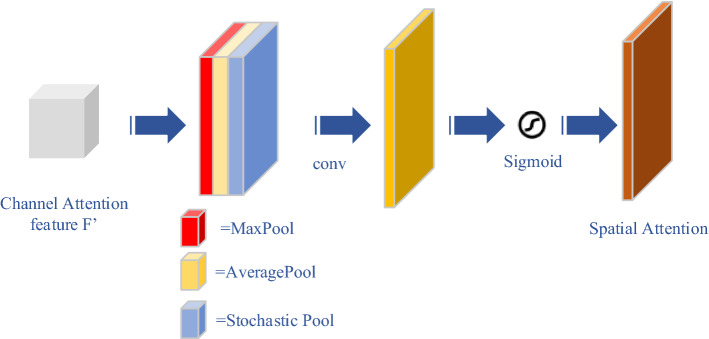
Structure of the spatial attention module. The stochastic pool function is also added to the average pool and the maximum pool functions, and then the three corresponding outputs are spliced together with the same dimension. Finally, the convolutional operation with a 7$\times$7 filter and the sigmoid function are utilized to obtain the screening features

*Spatial attention module:* the structure of the spatial attention module is illustrated in Fig. 9.

Different to the channel attention module, the spatial attention module focused on “where” is the information section to supply channel attention. Suppose the input of the spatial attention module is the vector $\mathbf {F} '$, which is the output of the channel attention module. And the overall procedure is calculated based on Formula (18)–(19).

18 $$\begin{aligned} \begin{aligned} M_s (\mathbf{F }')=\sigma (f ^{7\times 7}(&\text {AvgPool}(\mathbf{F }');\text {MaxPool}(\mathbf{F }');\\&\text {StoPool}(\mathbf{F }'))), \end{aligned}\end{aligned}$$

19 $$\begin{aligned} \mathbf{F }''=M_s (\mathbf{F }')\otimes \mathbf{F ,} \end{aligned}$$

where $f^{7\times 7}$() represents a convolutional operation with the 7$\times$7 filter.

## Data Availability

Not applicable.
